# Bag-in-the-lens technique

**DOI:** 10.3389/fopht.2026.1813018

**Published:** 2026-05-08

**Authors:** Shervine Ameli, Iva Krolo, Silke Oellerich, Luc Van Os, Marie-José Tassignon, Sorcha Ní Dhubhghaill

**Affiliations:** 1 Department of Ophthalmology, Universitair Ziekenhuis Brussel, Jette, Belgium; 2 Department of Medicine and Pharmacology, Vrije Universiteit Brussel (VUB), Brussels, Belgium; 3 Neuroprotection and Neuromodulation (NEUR) Research Group, Center for Neurosciences (C4N), Vrije Universiteit, Brussel (VUB), Brussels, Belgium; 4 Department of Translational Neurosciences, University of Antwerp, Wilrijk, Belgium; 5 Department of Ophthalmology, Antwerp University Hospital, Edegem, Belgium

**Keywords:** bag-in-the-lens, BIL, cataract surgery, intraocular lens, optic capture, posterior capsulorhexis

## Abstract

Optimal centration of an intraocular lens (IOL) is important for obtaining its intended visual properties. The bag-in-the-lens (BIL) approach is a surgical technique that optimizes centration by centring the lens using both an anterior and a posterior capsulorhexis. The BIL is a monofocal hydrophilic IOL with a biconvex optic and two elliptical plane haptics. The space between the haptics creates an equatorial groove. The IOL is then suspended by placing both anterior and posterior capsular bag rhexis openings into this equatorial IOL groove. As a result, the BIL IOL captures residual lens epithelial cells and therefore additionally seals its position avoiding opacification of the visual axis. In this review, we offer an overview of current literature on the BIL technique.

## 1 Introduction

Cataract extraction with intraocular lens (IOL) insertion is among the most frequently performed surgeries worldwide (1). Conventional surgery based on placing the lens in the bag, may lead sooner or later to posterior capsular opacification (PCO), reducing visual quality (2). Although PCO is easily treated with Nd: YAG laser capsulotomy, optimal quality of vision is often no longer obtained and additional complications such as retinal detachment (RD), increased IOP, cystoid macular edema, and uveitis can occur (3). Lens epithelial cells (LECs) can also undergo transformation which may lead to capsular fibrosis, which can deviate the initial lens position. To address these issues, the bag-in-the-lens (BIL) technique was introduced by Tassignon et al. in 2002, aiming specifically at preventing PCO (4). This review provides a comprehensive overview of the available literature on the BIL implantation technique, with a focus on surgical technique, clinical outcomes, and special indications.

## 2 Methods

A literature search was conducted in PubMed to identify publications on the BIL technique. The following query was used: “bag-in-the-lens” OR “bag in the lens” OR (“Tassignon” AND “BIL”). We also manually searched through similar articles offered by the database and reference citation analysis. We considered all publication years and included articles published up to January 14^th^ 2026. This search retrieved 59 results, after which titles and abstracts were screened for eligibility. Full texts (or abstracts when full text was unavailable) were reviewed for potentially relevant information. One additional article was found through reference citation analysis. The following exclusion criteria were implemented: articles in languages other than English or French (n = 2), non-original publications (review articles; n = 2), letters to the editor (n = 1), as well as publications outside of the scope of this review (n = 7). This resulted in a total of 48 articles which were included. Selected articles were analyzed and relevant information pertaining to predefined categories was extracted. These categories encompassed various aspects of BIL IOL implantation, including its historical context, underlying mechanisms, surgical techniques, clinical outcomes, and special indications for its use.

## 3 The bag-in-the-lens design and principle

### 3.1 Concept of posterior capsular opacification prevention

**Figure 1:**
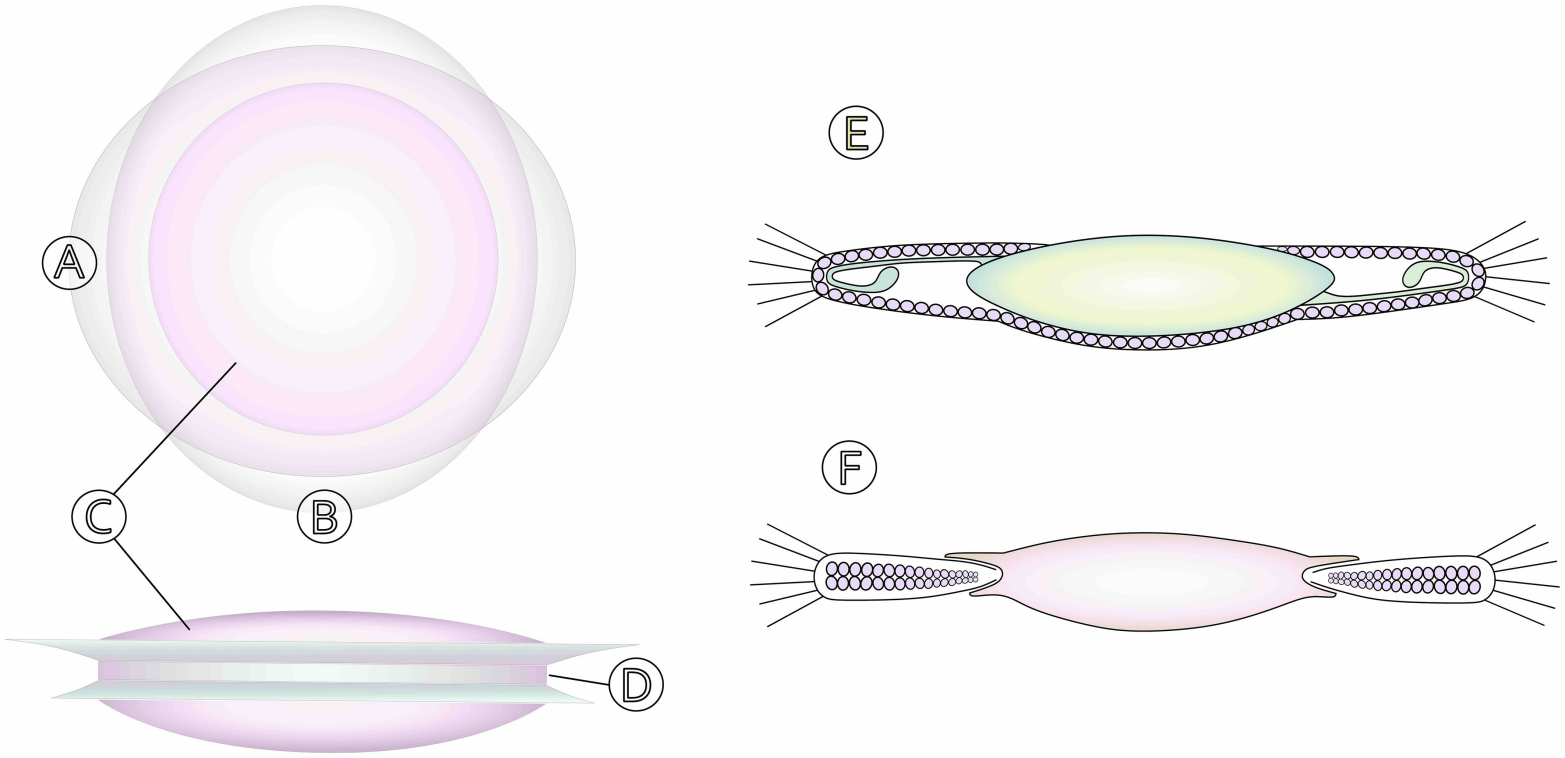
Schematic drawing of the BIL IOL design: **(A)** anterior haptic, **(B)** posterior haptic, **(C)** lens optic, **(D)** lens groove. Compared to the conventional in-the-bag IOL **(E)**, remnants of the anterior and posterior capsule are placed in the BIL IOL groove **(F)**, preventing the spread of LECs and PCO formation. Patent MJ B.R Tassignon, Intraocular lens and method for preventing secondary opacification, US Patent 6,027,531, Feb. 22, 2000.

The BIL IOL has a central 5-mm biconvex optic and a haptic design featuring anterior and posterior oval-shaped lips arranged perpendicularly, forming a central groove between them (**Figures 1A–F**). This configuration follows the position of the capsular bag and prevents IOL tilting or luxation into Berger’s space. While initially a rigid IOL crafted from PMMA by PhysIOL, Belgium (4), the BIL concept’s success led to the development of the foldable Morcher 89A lens (the current standard model). The Morcher 89A lens is made of foldable hydrophilic acrylic material (28% water content), and measures 7.5 mm at its widest and 6.5 mm at its narrowest point (5). Two modified versions of this standard model exist: a smaller type, the 89D, and a larger type, the 89F, where the front haptic measures 8.5 mm (Ekhardt modification) leading to increased stability in combined phacovitrectomy procedures.

**Figure 2:**
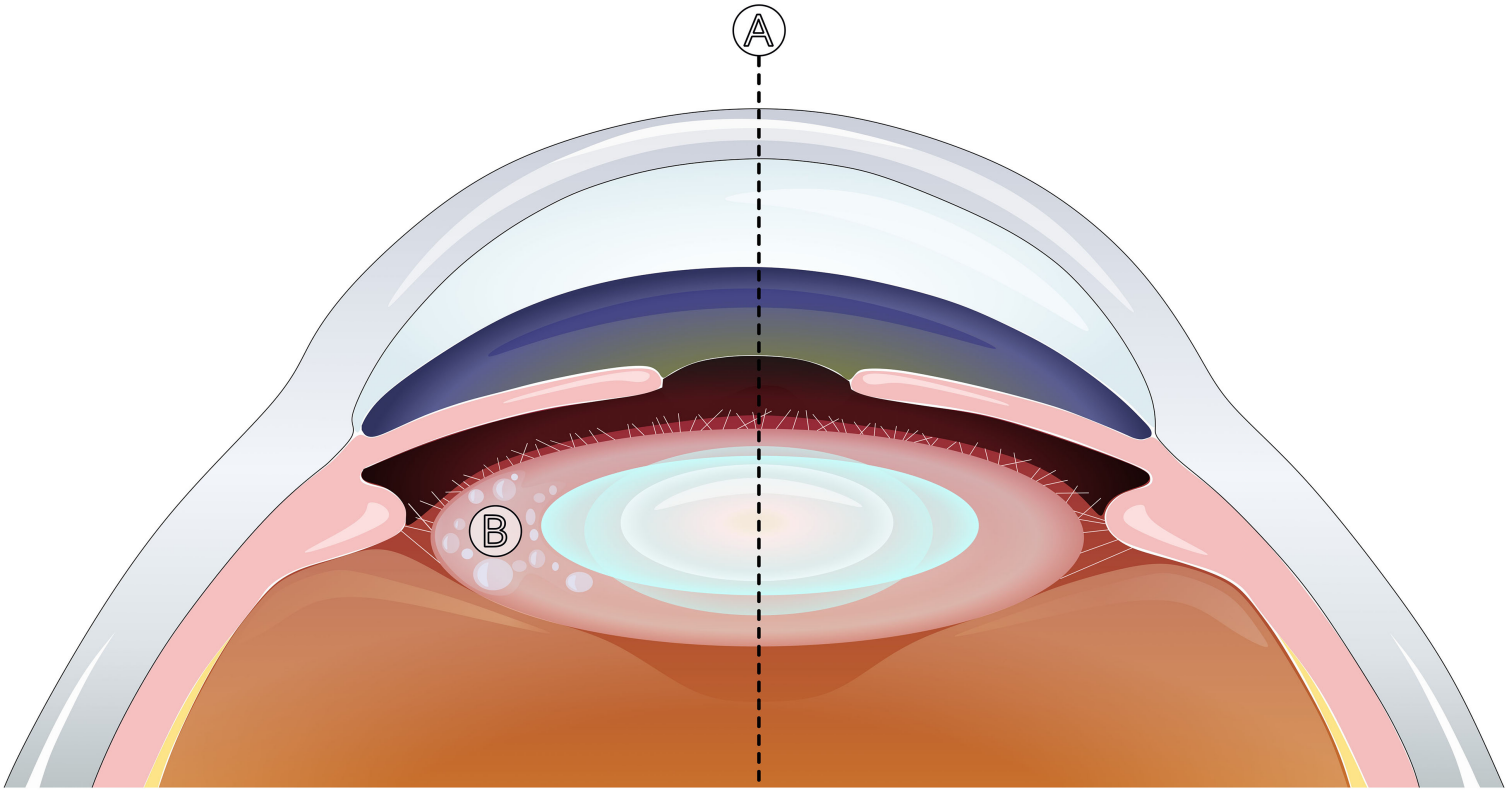
Optic capture with the BIL IOL. By placing the anterior and posterior capsular bag openings into the IOL groove, the BIL design enables an adequate optic centration at the visual axis **(A)** and prevents VAO by sequestering LECs **(B)**.

During surgery, calibrated continuous curvilinear capsulorhexes (CCCs) are created in both the anterior (ACCC) and posterior (PCCC) lens capsules. By ensuring two identically sized CCCs, both capsules are tightly fitted into the IOL’s peripheral groove, hence the term “bag-in-the-lens”. This technique is also referred to as the “twin-capsulorhexis” method (4). When both capsules are sited in the groove, residual LECs become confined within the remaining space of the lens bag, effectively preventing their migration toward the pupillary axis (**Figure 1F**). As a result, the visual axis remains clear (**Figure 2**) (4).

This PCO-prevention property was originally validated experimentally in living rabbit eyes, and postmortem human eyes (6–9). Furthermore, histopathologic evaluation of postmortem eyes has demonstrated the formation of “rhexis fibrosis”; fibrocellular tissue development along the anterior rhexis margin. This facilitates adhesion between both capsules within the IOL groove, contributing to postoperative IOL stability (6). Fluorophotometry studies have shown that there were no significant differences in fluorescein distribution patterns in eyes with or without surgically controlled PCCC, confirming that the anterior hyaloid, rather than the posterior capsule, maintains the anterior ocular barrier’s integrity (10).

### 3.2 Surgical technique

After preoperative dilation, a clear corneal incision is performed, followed by the instillation of a long chain dispersive ophthalmic viscosurgical device (OVD) (Healon GV^®^, Johnson &Johnson, USA). A 4.5 or 5.0 mm PMMA ring caliper (in pediatric or adult eyes, respectively) is placed on the surface of the anterior capsule, to guide and perform the ACCC using a capsulorhexis microforceps. A correctly sized and centered anterior continuous curvilinear capsulorhexis is essential so a ring caliper is used. Using the Purkinje reflexes or the mathematical centre of the limbus as centration parameter, proper centration of the ACCC is achieved. This can be assisted by a device known as an “Eye Cage” (5).

After removal of the ring caliper, phacoemulsification of the lens nucleus is performed, followed by aspiration of remaining lens cortex. The anterior chamber is then refilled with OVD, pressing anterior capsule remnants onto the posterior capsule, taking care not to fill in the capsular bag. The posterior capsule is carefully punctured centrally using a 27-gauge needle. A light molecular weight cohesive OVD (Healon Pro^®^, Johnson and Johson, USA) is injected beneath the posterior capsule into Berger’s space to push back the anterior hyaloid. The PCCC is then performed using the ACCC as a sizing guide.

After loading the BIL IOL into its cartridge, the lens is injected into the anterior chamber. The optic is gently pressed, to position the posterior haptic lip behind the posterior capsule, and the anterior lip in front of the anterior capsule, thereby securing both capsules within the IOL’s interhaptic (central) groove. OVD is intentionally left in the Berger’s space and removed in its entirety from the anterior chamber. To prevent iris capture, carbachol is administered at the end of the surgical procedure and the case is completed as normal.

Femtosecond laser capsulorhexis has been explored to enhance precision and reproducibility for the BIL technique (11), in performing both anterior and posterior capsulotomies (12). The method involves two separate laser treatments: the first for anterior capsulotomy, followed by manual lens removal, after which the posterior capsule is punctured and OVD is injected to push back the anterior vitreous surface, enabling the second laser treatment for posterior capsulotomy. It is important to note that a planned PCCC maintains physiological diffusion across the aqueous-vitreous interface, unlike accidental (or laser-induced) posterior capsule rupture, which may disrupt the barrier (13).

### 3.3 Special indications

#### 3.3.1 Paediatric cataracts

In very young children undergoing a LIB cataract procedure, PCO is nearly inevitable so performing a primary posterior capsulotomy together with an anterior vitrectomy before IOL implantation is standard practice (14). Should the primary posterior capsulotomy not be combined with an anterior vitrectomy, visual axis obscuration (VAO) occurs in up to 60% of cases. This is because the intact anterior vitreous functions as a scaffold across which LECs migrate, leading to VAO. However, VAO is still seen postoperatively even after anterior vitrectomy (perhaps due to insufficient removal of the scaffold) (14). Secondary cataract rates of 9.2% have been reported with these practices (15).

Avoiding an anterior vitrectomy can have a benefit since the anterior hyaloid maintains a barrier between the eye’s anterior and posterior segments (10). It is possible that an intact vitreous membrane plays a role in reducing the occurrence of postoperative complications, particularly secondary glaucoma, and postoperative inflammation (16). Vasavada et al. recommended PCCC with anterior vitrectomy only in children younger than 3 years (with a conventional in-the-bag IOL) (14). In children, the BIL IOL implantation technique does not include an anterior vitrectomy as standard except in cases of persistent fetal vasculature or anterior interface dysgenesis pathologies (17, 18).

The first results of BIL IOL implantation in paediatric patients were published in 2007 by the originating center and showed safety and maintenance of a clear visual axis (19). This was followed up by a larger prospective study which included 54 eyes of 37 children aged 2 months - 14 years (17). A clear visual axis was seen in all cases with follow-up of 8.3+/-4.9 years, provided that IOL insertion was appropriate (93.8% of all cases, 100% of cases older than 1 year of age). A subsequent study showed that if the BIL is placed in the anterior rhexis only (without posterior capture), VAO can still occur, thereby emphasizing the role of trapping the capsular cells in the groove. The results of Sand et al. in their paper on first experience with the BIL implantation in children additionally illustrates the importance of proper BIL IOL positioning to guarantee absence of VAO (20).

The first long-term follow-up results after paediatric BIL IOL implantation were published by Van Looveren et al., reporting on the outcomes of the BIL procedure in 133 cases with a 5 year follow up for 46 eyes (21). Four eyes had persistent fetal vasculature and underwent anterior vitrectomy routinely while no anterior vitrectomy was performed in the other eyes. In 91.3% of eyes clear visual axis was maintained over 5 years. A large Swedish cohort study published in 2018 included 109 eyes of 84 children with a median follow-up time of 2.8 years (7 months – 5.8 years) and reported a clear visual axis in 95.4% of cases (22). A further retrospective comparative cohort study (64 eyes in the BIL group and 50 eyes in the LIB group) reported significantly lower surgical reintervention rates (particularly for VAO) with BIL placement (17.1%) compared with LIB placement (38%) (23).

SRK/T remains the most widely used formula for BIL power calculation in paediatric cases, and at present no alternative formula has been specifically established as superior for BIL implantation in children. The difficulty of IOL power calculation in children lies mainly in the limited predictability of ocular growth, which varies with both age and the underlying cause of the congenital cataract. Lytvynchuk et al. described the accuracy of BIL IOL power calculation with the SRK/T formula in children and found that the prediction error exhibited an inverse correlation with age, peaking at 3.43D in the age group <3 months, and gradually declining in children aged >36 months (24). Moreover, eyes with an axial length <20.0 mm displayed a statistically significant higher prediction error (mean 2.67D) than eyes with an axial length >20.0 mm (mean 1.44D).

#### 3.3.2 Astigmatism correction using a toric BIL

Prior to implementation of a toric component within the optic of an IOL, it is essential to examine three key parameters of the respective monofocal IOL. These include surgically induced astigmatism (SIA), IOL rotation stability, and IOL centration stability (25). The amount of SIA after BIL IOL implantation was reported in 100 eyes of 58 patients and found to be negligible (26). To validate the stability of the BIL IOL over time, Verbruggen et al. performed a retrospective analysis on 180 eyes and showed minimal IOL deviation from the intended axis, with no decentration exceeding 0.8 mm. Importantly, there was no significant correlation between IOL decentration and visual outcome. No significant variation between the IOL position was observed after a one-year follow up, and BIL IOL implantation showed better centration in comparison to LIB acrylic IOL implantation (27). In contrast to conventional LIB implantation, where achieving surgeon-controlled centration within the capsular bag is not feasible, the BIL technique enables the surgeon to align the IOL precisely along the patient’s visual axis (28).

The first clinical results after implantation of spherotoric IOLs using the BIL implantation technique were published in 2011 (29). The results showed that spherotoric BIL IOLs performed comparably to other toric IOLs for astigmatism (30). However, patients with an irregular astigmatism exceeding 10° ought to be advised that the anticipated outcomes might be less reliable (29). Nonetheless, the toric BIL IOL allows rotation during a secondary surgery without encountering challenges related to haptics embedded within fibrotic proliferative capsule tissue (25).

Rozema et al. evaluated the postoperative IOL rotation (rotational stability) and concluded that the average rotation observed in the BIL IOL was about 1° less than rotation values documented for other IOL types (31). Furthermore, no correlation was detected between IOL rotation and IOL power, nor was a correlation found with patient age. The 89A toric BIL IOL features astigmatic markings etched on the side corresponding to the cylinder correction. If the toric BIL IOL should be inverted, the astigmatic effective refractive power will be slightly reduced, which effect can purposely be used in some conditions where the full toric power should be reduced.

#### 3.3.3 Additional lens fixation devices and techniques

In cases of important zonular or capsular instability, the BIL was originally contraindicated. To address this limitation, bean-shaped ring segments were developed to reinforce compromised capsular bags (32). Following lens removal, the bean segments are inserted into either the capsular bag or ciliary sulcus and oriented 180° apart. Upon BIL IOL insertion, the inner semicircles of the segments slot into the interhaptic groove, providing additional support. This combination of residual capsular support and ring segment reinforcement helps preventing IOL disinsertion and decentration (32, 33). For cases of severe zonulopathy where scleral fixation is required, modified bean-shaped ring segments have been developed. These segments feature an incorporated eyelet in the outer semicircle, allowing fixation to the sclera via sutures (34). In 2021, Van Os et al. reported on an additional adaptation for the use of bean-shaped segments in children with congenital ectopia lentis. In this technique, the complex of the capsular bag, BIL IOL and bean-shaped segments is further centrated and suspended by adding a 6–0 Polypropylene loop which is fixated to the sclera. This resulted in a stable and well-centered position of the IOL (35).

### 3.4 Bag-in-the-lens intraocular lens exchange

Although rare, opacification of hydrophilic acrylic BIL IOLs can develop years after implantation due to calcium and phosphate precipitate deposition (36–38). The first reported case of BIL IOL exchange for optic opacification was published by Ní Dhubhghaill et al. in 2015 (39). Compared to standard in-the-bag IOL exchange, which is often high-risk due to fibrotic adhesions between the haptics and capsular bag, BIL IOL exchange offers distinct advantages (40). The BIL technique minimizes contact between the IOL and capsule, allowing for easier explantation without disengaging the haptics from the bag. This reduces the risk of capsular rupture and facilitates implantation of a replacement BIL IOL (39).

Ní Dhubhghaill et al. described separation of the lens capsule from the inter-haptic groove by using an IOL rotator spatula (Bausch & Lomb) and an OVD cannula (39). The OVD cannula was then advanced underneath the IOL and OVD was injected into the Berger space to displace the anterior hyaloid. Subsequently, the IOL was completely separated from the capsule and moved into the anterior chamber. It was then grasped with coaxial forceps and extracted through the main incision without needing to cut the IOL. The most common intraoperative complication during IOL exchange procedures was vitreous prolapse, occurring in approximately 16% of cases (41).

Another report of BIL IOL opacification described a wave-like calcium deposition on the posterior surface of the lens (42). The explanation given involved the abnormal fluid flow and stagnation in an unusual retrolenticular space of a highly hyperopic eye.

## 4 Clinical outcomes

### 4.1 Lens stability over the long term

Ensuring optimal centration of the IOL is important for obtaining optimal visual outcomes. The incidence of LIB IOL dislocation is around 0.2-3% (43–46), mainly in cases of prior vitreoretinal surgery, trauma, and systemic connective tissue or ocular disorders such as pseudoexfoliation syndrome, that can lead to zonular weakness. Postoperatively, IOL decentration may occur due to PCO and capsular contraction. PCO incidence ranges from 5% up to 50% in risk-related cataract surgeries, as in pediatric cases, traumatic cataracts, inflammation, diabetes, or complicated surgeries (47).

Most of these complications can be avoided while simultaneously obtaining a strong and stable optic capture, by using the BIL implantation technique (48). The stable IOL suspension is obtained by placing both anterior and posterior capsular bag openings into the BIL IOL groove (**Figure 2**). Structurally, the BIL IOL isolates residual LECs and therefore additionally seals its position, simultaneously avoiding visual axis PCO formation.

Werner et al. analyzed the clinical and histopathologic features of 6 eyes obtained postmortem and implanted with the BIL (6). The development of ‘rhexis fibrosis’ was observed; formation of fibrocellular tissue on the inner surface of the anterior rhexis margin. Rhexis fibrosis should facilitate adhesion between the anterior and posterior capsules within the IOL groove and likely contributes to improved IOL stability postoperatively (6). There were no signs of capsular bag distortion, and proliferative/regenerative material remained sequestered to its equatorial intercapsular region, while the optic area remained clear. The authors concluded that BIL IOL centration and postoperative stability depend primarily on the performance of centered anterior and posterior capsulorhexes of the appropriate size. These results confirm the findings of other work that found the BIL IOL centration to be unchanged 5 weeks, 6 months, and 1 year postoperatively, demonstrating it is not impacted by capsular healing over time (27).

### 4.2 Visual axis opacification and PCO prevention

De Groot et al. reported a complete absence of LEC proliferation on the optic during an average follow-up of 22.7 months (range 12 to 64 months) in 63 eyes (55 patients; 7 children and 48 adults) undergoing cataract surgery with BIL IOL implantation. All eyes maintained a clear visual axis, with LEC growth restricted to the peripheral capsular bag (49). Studies that confirmed the presence of visual axis opacification (VAO) in adulthood after BIL implantation were limited only to two cases of opacification of the optic itself (39, 42). Studies reporting on the incidence of VAO after BIL IOL implantation in children show varying rates (16, 21, 22, 50, 51). Depending on the term of follow-up in infants, VAO rates varied between 5.6% (50) and 8.7% (21).

Comparative studies further support the BIL design’s effectiveness in PCO prevention. In a series of 45 BIL and 55 LIB eyes undergoing phaco-vitrectomy, no PCO occurred in the BIL group, while 27 LIB eyes developed PCO (p<0.001) of which 20 required laser capsulotomies (52). Similarly, Leysen et al. compared cumulative Nd: YAG laser capsulotomy rates between the Morcher 89A (BIL) and Morcher 92S (LIB) IOLs (both made from the same biomaterial) implantation (28). Among 100 eyes per group, the LIB group showed a 28% capsulotomy rate after 71 months, versus no cases in the BIL group.

### 4.3 Visual performance

According to reports on paediatric cataract cases, refractive outcomes after 5 years were found to be satisfactory when employing the SRK/T formula (21). Fifty-two percent of eyes, all age groups confounded, showed spherical equivalent within 2.00D of emmetropia and all refractive errors improved with spectacles or contact lenses (21). Visual acuity after BIL IOL implantation in combined vitrectomy-lensectomy procedures did not significantly differ when compared to the LIB implantation technique (p=0.74), showing the mean gain in the visual acuity of -0.52 and -0.56 logMAR, for the BIL and LIB procedures, respectively (53).

The capsular dependent suspension of the lens by the bag has led to the speculation that it may enable postoperative pseudophakic accommodation. Theoretically, the capsular fixation could allow forward axial movement propelled by ciliary muscle contraction (54, 55). A 2010 randomized clinical trial compared the accommodative performance of the BIL IOL with the standard LIB IOL implantation technique in presbyopic patients and found negligible axial movement of both IOL types during near visual effort (56).

A retrospective analysis of patients implanted with multifocal sulcus add-on IOLs with BIL as a primary IOL demonstrated a higher incidence of dysphotopsia in comparison to previous reports, as well as a higher incidence of pigmented IOL deposits on the add-on IOL (57). Dysphotopsia was the primary reason for add-on IOL explantation in this cohort.

BIL has shown a good visual performance in special cases like megalocornea with white-to-white extremes of 16 mm. Out of the 3 eyes that had BIL IOL implantation, 1 resulted in hypermetropic refractive outcome (58), while 2 reached emmetropia (58, 59).

### 4.4 Adverse events

A prospective consecutive cohort study regarding the clinical outcomes and adverse events associated to the BIL IOL implantation technique, compared with the International Organization for Standardization (ISO) standard 11979-7:2006, was published in 2011 (51, 60).

#### 4.4.1 Iris complications

In iris-related complications, most reports referred to BIL pupil incarceration and intraoperative small pupil management where the iris is captured in the groove making this a specific complication associated with the BIL. Most cases of BIL pupil incarceration were managed with mydriatic drops, but in up to 7.3% of cases surgical repositioning, explantation, or exchange was required (22, 29, 50, 51). It has been previously reported that luxation occurs in cases of oversized anterior and/or posterior capsulorhexis openings (16, 19, 21, 50). Tassignon et al. demonstrated iris capture incidences of 2.35%, with 15 out of 19 eyes experiencing it during the early postoperative phase, while in 4 eyes this complication occurred in the later postoperative course (51). The use of a miotic at the end of the surgery can further decrease the risk of iris incarceration.

Theoretically, the BIL IOL implantation technique prevents the inflammatory response associated with LEC release into the aqueous humor, thereby aiding in posterior synechiae prevention (61). Furthermore, microtrauma-induced inflammation is minimized because BIL IOL implantation involves no contact with the sulcus and ciliary body (21). When comparing BIL and LIB IOL implantation techniques in combined phaco-vitrectomy surgery, posterior synechiae have been found in 40% of LIB eyes compared to 2% in the BIL group (52).

#### 4.4.2 Retinal detachment

As per ISO standards, the threshold for RDs within 12 months of follow-up is 1.80%. A 2011 report observed an overall RD incidence of 1.24% after BIL IOL implantation. However, looking at the cohort’s 12-month subset specifically, 4 RD cases occurred, corresponding to a 0.90% incidence rate (51).

In a 2015 prospective cohort study, Tassignon et al. reported rhegmatogenous RD post-BIL IOL implantation, with an incidence of 0.49% at 1 year and 0.84% at 2 years (62). Notably, comparative studies of rhegmatogenous RD after LIB IOL implantation often exclude patients with significant risk factors (63, 64), which may lead to underestimation of RD rates after LIB implantation. In the same cohort, after excluding high-risk patients, no RD occurred within 1 year and one RD occurred within 2 years (0.15% incidence) (62).

This patient cohort was extended in 2020, additionally identifying risk factors significant for postoperative RD (65). The patient cohort showed a relatively young mean age (67.5 ± 16.1 years), high prevalence of high myopia (16.7%), and 61 eyes with a history of contralateral RD. The study observed RD in 36 out of 3385 eyes (1.06%) and the following cumulative incidences were noted: 0.35% over 1 year, 0.66% over 2 years, 1.17% over 5 years. Despite the relatively high prevalence of risk factors within this study population, RD rates were comparable to, and in some cases slightly lower than incidences reported with conventional LIB IOL implantation (66, 67).

Beyond incidence reporting, the vitreolenticular interface may be relevant to pseudophakic posterior-segment events. Vael et al. showed with intraoperative OCT that controlled posterior capsule puncture and OVD inflation of Berger’s space enables PCCC with a very low incidence of anterior hyaloid damage. They also hypothesized that inflating Berger’s space partially compensates for the volume loss after lens removal, resulting in minimal immediate postoperative anterior displacement of the anterior vitreous. Whether this reduces posterior-segment complications remains to be confirmed (18).

#### 4.4.3 Macular edema

In 2020, Scheers et al. conducted the first in-depth analysis on the incidence of clinically significant pseudophakic cystoid macular edema (CSPME) following BIL IOL implantation (68). This study cohort was later expanded to 2419 first-operated eyes (47). The 3-month CSPME incidence remained at 0.00% in patients without risk factors and reached 0.57% in those with identified risk factors (diabetes, wet age-related macular degeneration, macular traction), resulting in an overall 3-month CSPME incidence of 0.29%. This study also identified significant risk factors for CSPME development: exudative age-related macular degeneration, retinal vein occlusion, and renal insufficiency (47).

#### 4.4.4 Intraoperative complications

There were several studies describing intra- and postoperative complications of the BIL IOL implantation technique in children of varying age groups (16, 20–22, 50, 69–71). The occurrence of secondary glaucoma of 13.8% found by Nyström et al. was notably higher than the 2.2% reported by Van Looveren et al. (21, 22). The disparity in glaucoma rates stems from the age variation of patients between the studies (2 weeks versus >2 months, respectively), with Nyström reporting a much lower glaucoma rate of 6.7% in children who were between 5 weeks and 2 years of age at the time of surgery. The association between younger age at surgery and an increased risk of secondary glaucoma, subsequently affecting visual outcomes, has been reported previously (72, 73). Reported incidences in comparable cohorts range from 12-13% (74). Nyström et al. also pointed out that most eyes developing glaucoma had concurrent ocular or systemic disease (22).

In a retrospective cohort study from a Norwegian center, Sand et al. analyzed results in 13 eyes with congenital cataract in infants <12 weeks of age and a median age at surgery of 28 days (20). The study found the BIL IOL technique to behold less intra-operative complications than the LIB implantation technique (70, 71). Because of vitreous prolapse or anterior vitreous opacities, anterior vitrectomy was performed in seven eyes. These results are consistent with previously performed studies (50, 69). Despite all eyes in this study presenting with a mild degree of persistent fetal vitreous, it is worth noting that six of them did not necessitate anterior vitrectomy. This suggests that anterior vitrectomy is not imperative in every instance of BIL IOL implantation during paediatric cataract surgery.

## 5 Conclusion

The current literature review provides an insight into the intraoperative particularities of the BIL technique, its special design and clinical indications, as well as postoperative outcomes. Due to its specific optic capture, the BIL IOL implantation procedure enables a clear visual axis, controlled IOL optic centration, and straightforward exchange with good postoperative predictability and refractive outcome. A limitation of the current review is that the published evidence remains weighted toward the originating group. Broader uptake of the BIL technique may be influenced by its surgical learning curve, particularly the need for accurate sizing and centration of the anterior and posterior continuous curvilinear capsulorhexes and familiarity with optic capture maneuvers. However, the available reports from independent external centers, particularly Scandinavian groups involved in pediatric cataract surgery, are broadly supportive of the technique. Further multicenter comparative studies will be valuable to confirm its generalizability across broader surgical settings.
